# Exploration of the Role of Vitamins in Preventing Neurodegenerative Diseases: Comprehensive Review on Preclinical and Clinical Findings

**DOI:** 10.2174/011570159X327677240902105443

**Published:** 2024-11-21

**Authors:** Liza Changkakoti, Rajan Rajabalaya, Sheba R. David, Ashok Kumar Balaraman, Hemalatha Sivasubramanian, Ashis K. Mukherjee, Asis Bala

**Affiliations:** 1 Pharmacology and Drug Discovery Research Laboratory, Division of Life Sciences; Institute of Advanced Study in Science and Technology (IASST), Vigyan Path, Guwahati, PIN-781035, Assam, India;; 2 Academy of Scientific and Innovative Research (AcSIR), AcSIR (an Indian Institute of National Importance), Sector 19, Kamla Nehru Nagar, Ghaziabad, Uttar Pradesh PIN-201002, India;; 3 PAPRSB Institute of Health Sciences, Universiti Brunei Darussalam, BE 1410 Bandar Seri Begawan, Brunei Darussalam;; 4 School of Pharmacy, University of Wyoming, Laramie, Wyoming, 82071, USA;; 5 Research Management Unit, University of Cyberjaya, Persiaran Bestari, Cyber 11, 63000, Cyberjaya, Selangor, Malaysia;; 6 Department of Pharmacognosy, Sri Ramachandra Faculty of Pharmacy, Sri Ramachandra Institute of Higher Education and Research (Deemed to be University), Porur, Chennai, India;; 7 Microbial Biotechnology and Protein Research Laboratory, Division of Life Sciences; Institute of Advanced Study in Science and Technology (IASST), Vigyan Path, Guwahati, PIN-781035, Assam, India

**Keywords:** Neurodegenerative diseases, vitamin, Parkinson's disease, Alzheimer's disease, Huntington's disease, multiple sclerosis, amyotrophic lateral sclerosis

## Abstract

Neurodegenerative diseases (NDDs) are a multifaceted and heterogeneous group of complex diseases. Unfortunately, a cure for these conditions has yet to be found, but there are ways to reduce the risk of developing them. Studies have shown that specific vitamins regulate the brain molecules and signaling pathways, which may help prevent degeneration. This review focuses on examining the role of vitamins in preventing five significant types of neurodegenerative diseases, including Parkinson's disease (PD), Alzheimer's disease (AD), Huntington's disease (HD), Multiple Sclerosis (MS), and Amyotrophic Lateral Sclerosis (ALS). This review also highlights promising and controversial findings about the potential impact of vitamins on this group of diseases. Several developed countries standardize daily dietary vitamin intake to meet nutrient requirements, improve health, and prevent chronic diseases like NDDs. However, more research is necessary to gain a more comprehensive understanding of their therapeutic benefits, including studies exploring different drug-dose paradigms, diverse humanized animal models, and clinical trials conducted in various locations.

## 1 INTRODUCTION

Micronutrients are known to play a vital role in the body's metabolism. Over time, there has been a growing interest in the importance of micronutrients for maintaining good health and preventing various diseases [1]. It is worth noting that the quantity of micronutrients in the human body is relatively low, with trace elements measuring up to 50 mg/kg and plasma levels of trace elements and vitamins ranging from µmol/L to mg/L [2]. Micronutrients, known as vitamins, play a critical role in the human body's growth, development, and normal functioning [3, 4]. These organic compounds regulate various biochemical processes [4]. Since the body cannot synthesize them sufficiently, they must be obtained from external sources, such as food [4]. Deficiencies in vitamins, particularly prolonged ones, can result in well-known diseases like Scurvy, Pellagra, Anaemia, Haemolytic Anaemia, and Osteomalacia [3]. Furthermore, recent studies have linked vitamin deficiencies to other diseases, including neurodegenerative diseases, and their impact on cognitive performance [3]. It is important to note that vitamin deficiencies are common among both infants and the elderly [4].

As the population ages, we are seeing a rise in age-related disorders, with neurodegenerative diseases being a significant concern [5]. For instance, The Lancet reported that in 2019, 57.4 million people were living with dementia (often seen in patients with Alzheimer's disease), and this number is expected to soar to 152.8 (130.8-175.9) million by 2050 [6]. These increasing cases bring significant economic and public policy burdens, as well as placing a strain on caregivers [5, 7]. The healthcare system is also heavily impacted, with multiple doctor visits, therapies, medications, and associated co-morbidities such as depression and dementia [8]. A growing body of evidence underscores the critical importance of nutrition in the development and prevalence of brain-related disorders. Specifically, micronutrient levels are known to significantly impact antioxidant enzyme activity, which can lead to DNA, protein, and fatty acid oxidation and crosslinking and depletion of mitochondrial ATP. Ultimately, these factors can contribute to the development of neurodegenerative diseases [7]. Studies have reported vitamins' preventative and protective activity against the pathologies of different neurodegenerative diseases. Interestingly, some studies have reported otherwise [4]. This review will delve into the intricate role of vitamins in preventing neurodegenerative diseases. Specifically, we will be focusing on five significant diseases: Parkinson's disease (PD), Alzheimer's disease (AD), Huntington's disease (HD), Multiple Sclerosis (MS), and Amyotrophic Lateral Sclerosis (ALS). The review aims to provide a detailed analysis of the latest updates on this topic, presenting them through several schematic and tabular representations for ease of understanding. By the end of this review, readers will have a comprehensive understanding of the impact of vitamins on preventing neurodegenerative diseases.

## 2 ROLE OF VITAMINS IN THE BODY

Micronutrients, such as vitamins, are essential for regulating a wide range of bodily functions [3]. They are involved in the growth and specialization of tissues, the processing of minerals, and the support of enzymes as co-factors or precursors [3]. They are also involved in enzyme systems and possess antioxidative activity that neutralizes free radical Reactive Oxygen Species (ROS) and Reactive Nitrogen Species (RNS) [3, 4]. Additionally, they act as modulators of cellular immunity and aid in wound healing, directly or by using substances created through their induction (cathelicidin LL-37). Vitamin C, D, Se, and Zn are micronutrients crucial in wound healing [2]. A healthy population's balanced diet should ideally provide the necessary vitamins to prevent various diseases. This highlights the importance of different types of vitamins in maintaining good health and preventing illnesses [4]. Table **1** presents various types of vitamins. Water-soluble vitamins are not stored in the body, so the body needs a continuous supply from outside sources. Fat-soluble vitamins, on the other hand, are stored in the body and dissolve in fats and oils. They are absorbed in the intestine.

### 2.1 Vitamin-like Micronutrient Intake Recommendations

Countries have established standard recommendations for micronutrient intake in regular diets based on studies of healthy populations. Deviations from these recommended intakes may indicate deficiency or potential toxicity [1]. For instance, the USA uses Dietary Reference Intakes (DRI), and the UK uses Reference Nutrient Intakes (RNI) as guidelines [1]. The main concern is whether a healthy diet provides sufficient nutrients [1]. Inadequate nutrient intake can result from socioeconomic constraints or increased requirements, such as during pregnancy, smoking, or illness recovery. Such groups may supplement their diet with additional nutrients to compensate for the deficiency. However, deficiencies can lead to adverse clinical impacts, such as metabolic and biochemical disturbances, infections, cognitive impairment, and exacerbation of existing diseases [1]. It is crucial to be aware that taking too many supplements can be harmful, especially for individuals who do not actually need them [1, 2]. It is crucial to note that Vitamins A, D, E, and K are fat soluble, meaning that they can be stored in the body. High doses of these vitamins can lead to toxicity [1]. Therefore, it is crucial to accurately identify the groups of people who need specific micronutrient amounts and provide appropriate dosage recommendations to address their inadequacy [1].

In countries like the U.S., which have advanced nutritional standards, the Dietary Reference Intakes (DRIs) for vitamins are established by the National Academy of Medicine (formerly the Institute of Medicine). Additionally, the WHO Library Cataloguing-in-Publication Data provides guidelines for the recommended daily intake of vitamins for both males and females, revealing notable differences between the genders, as shown in Fig. (**1**).

## 3 ROLE OF VITAMINS AND MICRONUTRIENTS IN THE NERVOUS SYSTEM

Research has shown that micronutrients, particularly vitamins, are crucial for the growth and development of neurons and for regulating their biochemical activities, ultimately impacting their overall functioning [3]. Vitamins play various roles in the nervous system, such as supporting neuronal survival, neurogenesis, and transmission. When deficient, they can lead to abnormal brain functioning, inducing mitochondrial dysfunction, oxidative stress, excitotoxicity, proteinaceous plaque deposition, and neurodegeneration [3]. Research has shown that vitamins impact cognitive performance [3]. Some vitamins are known to reduce the levels of oxidants and ROS in the body. A deficiency of these vitamins, indicated by higher homocysteine levels, can lead to memory impairment [3]. Certain studies have reported that vitamins may have a preventative effect against specific neurodegenerative diseases such as Alzheimer's and Parkinson's disease. They may also have therapeutic properties for various neurodegenerative diseases, including Huntington’s disease, prion disease, and Multiple Sclerosis [4]. Fig. (**2**) provides a concise visual overview of the distinctive attributes, significant indicators, and symptoms of each disease, as well as their underlying causes. Factors such as reactive oxygen and nitrogen species (ROS, RNS), the impact of neurotoxic oligomers and heavy metals, and a solid genetic predisposition all play a crucial role in developing NDD.

Although there is an awareness of the potential therapeutic benefits of vitamins, their usage has not yet been widely encouraged due to a lack of evidence demonstrating their ability to penetrate the blood-brain barrier. It is worth noting that the physicochemical properties of vitamins play a significant role in determining their ability to cross this barrier [3]. While specific studies have suggested that vitamins may prevent amyloid-beta and tau pathology, other research has reported no significant correlation between vitamin activity and the prevention of neurodegenerative diseases [4]. The disruption of micronutrient levels can cause a range of issues in the nervous system, including damage to the peripheral nerves from demyelination or axonal damage, damage to the central nervous system, and a specific form of myeloneuropathy that affects both. Such dysregulations can result in pathological changes during nervous system development and give rise to what are known as “nutritional” neuropathies [2].

Research has shown that incorporating essential nutrients such as vitamins, long-chain-polyunsaturated fatty acids, and minerals into one's diet can reduce inflammation, boost antioxidative activity, and decrease the risk of age-related illnesses, including neurodegenerative diseases. Studies have indicated that this dietary supplementation has positively affected patients with Mild Cognitive Impairment (MCI). Nevertheless, it is worth noting that weaker outcomes have been observed in patients in advanced stages of neurodegenerative diseases [7]. Neurodegenerative diseases such as Alzheimer's, Parkinson's, Lewy Body Dementia, and Vascular Dementia can lead to Cognitive Impairment. These disorders share common features, including the abnormal buildup of proteins in the brain and spinal cord, neuroinflammation, glial activation, and changes in metabolic function within both the central and peripheral nervous systems [7]. Furthermore, according to recent reports, n-3 Polyunsaturated fatty acids can help maintain cognitive function and promote the growth of new neurons in adults. They do this by modulating the remodeling of cell membranes, reducing oxidative stress, and regulating inflammatory mediators. This suggests that nutritional substances with anti-inflammatory and antioxidant properties, which can cross the blood-brain barrier and target specific cells, could be used as therapeutic agents to manage neurodegenerative diseases [7].

## 4 ROLE OF VITAMINS AND MICRONUTRIENTS IN VARIOUS NEURODEGENERATIVE DISEASES

### 4.1 Alzheimer’s Disease

Alzheimer's is a prevalent, progressive neurodegenerative disease that commonly causes dementia in the elderly population [3, 4]. It is the most common neurodegenerative disease worldwide [4]. This disease can impair a person's ability to learn and remember and their capacity to conduct daily activities [3]. The gradual deterioration of neurons identifies Alzheimer's neuropathology due to the buildup of amyloid (Aβ) plaques outside the neurons and neurofibrillary tangles (NFT) of the hyperphosphorylated tau protein inside the neurons [3, 4]. Oxidative stress is a leading cause of neurodegeneration in AD [4]. A vitamin deficiency is one of the numerous hypotheses believed to contribute to the aetiology of AD [3]. Vitamins are utilized as adjuvants in the therapeutic regime of AD owing to their anti-oxidative activity [4].

Maintaining the integrity of the myelin sheath in neurons means ensuring that the protective covering around nerve fibers in the brain and spinal cord remains intact and functional [9]. It is extremely important to preserve the integrity of the myelin sheath in neurons, as it serves a vital function in protecting and insulating these cells [9, 10]. Recent publications have shown that a lack of vitamin B12 can lead to the destruction of the myelin sheath, emphasizing the critical role of this vitamin in maintaining neurological health [9]. Vitamin C deficiency disrupts the integrity of the BBB. Oxidative stress in the neurons *via* a decline in expressions of the antioxidant molecules in the mitochondria [10]. Furthermore, vitamin C deficiency causes neurodegeneration. However, it enhances the formation of Aβ plaques between neurons and the phosphorylation of tau proteins in microtubules to form neurofibrillary tangles [11]. Very low levels of Vit D were observed in late Alzheimer’s patients with non-carrier ApoEɛ4 [12].

Table **2** [13-27] and Fig. (**3**) represent the results of various preclinical studies examining the effects of vitamins on Alzheimer's disease. The table includes details on the type of study, the outcomes, and the study's significance, all obtained from PubMed. Vitamin A inhibits the Aβ aggregation with two Aβ fragments, Aβ1-16 and Aβ25-35, and reduces the toxic B-amyloid fragments and neuronal dysfunction in experimental animal models [13, 14]. In a rat model of Alzheimer's disease, supplementation with ascorbic acid at 100 mg/kg for 15 days improved biochemical and behavioral deficits and reduced neuropathological changes due to its antioxidant properties [17]. Vit D was found to Improve age-related cognitive decline by reducing antioxidative potential and increasing amyloid-beta production [20-22]. Furthermore, multivitamins, including Vitamins B6, B12, folate, and choline, are also found to protect the brain in low-oxygen conditions and improve memory [25-27].

Table **3** [28-55] presents the results of clinical studies on vitamins for Alzheimer's Disease. The data was collected from PubMed, including the study type, outcomes, and significance. It is well reported that the levels of Vit A, Vit B, and Vit C in the blood serum of AD patients were found to be low when compared to healthy individuals (with intact neurocognitive function). The regulatory role of Vit C, Vit D, Vit E, and Vit K in AD patients was reported in many pieces of literature [16, 29, 30]. A combination of vitamin C and vitamin E supplementation reduced the prevalence and incidence of AD, as evidenced by a recent report [34]. Several types of B vitamins (Vit B9, Vit B12, Vit B6, Vit E, and Vit B2) are found to be involved in the metabolism of homocysteine and Retinoids; Vit E and Vit B supplements improve the cognitive function of AD patients [35, 37-39].

### 4.2 Parkinson’s Disease

Parkinson's disease is a common affliction among the elderly, and it occurs when dopaminergic neurons in the midbrain region degenerate. This condition can lead to several both motor and non-motor symptoms. Unfortunately, there are currently no effective therapies available that can prevent the onset of Parkinson's or halt its progression. The currently used treatments only manage the disease's symptoms, making it essential to seek out alternative therapies with fewer side effects [4]. Vitamins exhibit therapeutic activity against Parkinson's pathology due to their anti-oxidative and anti-inflammatory effects [4].

Vit A involves multiple signaling pathways that regulate gene expression, neural cell differentiation, and patterning in neural tube formation. It has also anti-oxidative solid properties [56-58]. Supplementation of thiamine was responsible for the delay in the progression and death of dopaminergic neurons in PD. Deficiency of thiamine causes death of the dopaminergic neurons in PD [59]. Vit B2 (*i.e*., Riboflavin) shows neuroprotective effects in oxidative stress, mitochondrial dysfunction, and glutamate excitotoxicity [60]. Furthermore, it activates pyridoxine for the metabolism or breakdown mechanism of homocysteine in the brain of Parkinson's patients [61]. A recent study revealed that Vit C showed neuroprotection against glutamate-induced neurodegeneration [62]. It also decreases the dopaminergic neuronal differentiation and causes oligomerization *via* overexpressing the α-synuclein in post-translational alterations in the neurons [63]. In contrast, humans show a dose-response towards vitamin C as high levels can be toxic [64-66]. On the other hand, an imbalance in Vit D homeostasis can lead to the accumulation of synuclein and disruption in the neuronal transmissions in the substantial nigra and pars compacta regions of the brain [67].

Receptor-based therapeutic approaches found Retinol beneficial in preventing the progression of PD in preclinical animal models, as shown in Table **4** [68]. Lycopene (Carotenoid) is a Vitamin A precursor that reverses physiological anomalies, oxidative stress, neurochemical abnormalities, and apoptosis. It also shows anti-apoptotic and antioxidative properties in this PD mouse model [69, 70]. In the UCH-L1 gene knockdown Drosophila PD models, the dose-dependent preventive effects of vitamin C were observed [71, 72]. Another fat-soluble Vitamin D significantly improved PD-like symptoms and associated pathology regarding mitochondrial abnormalities and synaptic impairment in a PD knockdown mouse model [73]. Whereas the chronic intake of VitE (500 mg/kg diet) lowers the death of dopaminergic neurons in the substantia nigra of the zitter mutant rat model of PD [74, 75]. Therefore, the preclinical finding also demonstrated the preventive role of various vitamins for PD, as shown in Table **4** [68-78] and Fig. (**3**).

Table **5** [79-92] depicts the clinical findings on vitamins in PD prevention. However, a Singaporean Chinese cohort-based study found no correlation between dietary antioxidants, such as carotenoids and vitamins (vitamins A, C, and E), and the risk of developing PD [80]. However, ascorbic acid (200 mg) significantly improves levodopa's absorption (100 mg levodopa and 10 mg carbidopa) in elderly PD patients [85]. Interestingly, Vit D and Vit E showed an inverse relation between their intake and PD occurrence as studied in a center study and meta-analysis on PD patients [74, 88, 89]. Furthermore, a multivitamin strategy consisting of Vitamins E, C, and carotenoids similarly does not appear to reduce the risk of Parkinson's disease [92].

### 4.3 Huntington's Disease

According to recent studies [4], a disease affecting the central nervous system is characterized by a progressive loss of GABA in the putamen region of the brain. This can lead to emotional problems and uncontrolled movements, among other symptoms [3]. HD shares similarities with AD and PD and is mainly characterized by dementia, motor abnormalities, and psychiatric problems [4]. The disease is caused by a genetic mutation that lengthens a section of the huntingtin gene, repeating trinucleotide (CAG) segments (more than 36 repeats). This can lead to toxic intracellular polyglutamine aggregates and neuronal degradation [4]. It has been suggested that vitamin level changes may play a role in HD pathogenesis [4].

Vitamin B3 improved motor functioning and prevented the progression of 3-nitro propionic acid (NPA)-induced HD-associated neurodegeneration by balancing the redox state in a rat model [93]. In addition, Vit B3 also improved histopathological parameters by decreasing the expression of lactate dehydrogenase, a marker of tissue degradation, and exhibited potent neuroprotective activity [93]. Supplementation of Vit D significantly improved clinical symptoms and augmented the life span of experimental animals suffering from HD [94]. Similarly, studies proved the synergistic effects of CoQ10 and VitE in the NPA-induced rat model of HD by reducing energy homeostasis [95]. On the other hand, CoQ10, ATP, and the electron transport chain activity were reduced. Interestingly, supplementation of CoQ10 and VitE reversed these abnormalities [95]. α-tocopherol, a significant constituent of Vit E, slowed the progression of motor abnormalities associated with HD, as evidenced in a clinical trial in Table **6** [93-96].

## 5 MULTIPLE SCLEROSIS

Multiple sclerosis is a chronic neurological disease that causes inflammation in the central nervous system, according to research by Kumar *et al*. (2021) [3]. This condition occurs when the immune system attacks the axons in the CNS, destroying myelin and axons and causing permanent disability. Progressive neuroinflammation is a defining characteristic of MS, which is then followed by neurodegeneration. Individuals with MS commonly experience problems with movement, vision, pain, and fatigue. While the exact cause of MS is unknown, both environmental and genetic factors are thought to contribute to its development [4].

Research on the impact of vitamins on multiple sclerosis in animals found that administering Vitamin C directly into the hippocampus of MS rats improved their memory during passive avoidance learning [97]. However, Vitamin C can lead to neuron demyelination by interfering with collagen synthesis [97]. On the other hand, Vitamin D supplementation was more effective in reducing MS-like neuroinflammation [98].

Clinical pieces of evidence, as shown in Table **7** [97-112], showed that Vit A exhibited anti-inflammatory and antioxidant activity in the brain, improved the function of astrocytes, and ameliorated the progression of MS [78, 99, 100-102]. In contrast, vitamin B plays a contradictory role, as some studies found no direct relation between Vit B levels and MS. Still, some clinical studies proved that high-dose thiamine (Vit B1) therapy improved the fatigue commonly associated with MS [103, 104]. Vit C and Vit D are found to have a preventive role in ameliorating MS-like neuroinflammation [98, 105-107, 112].

## 6 AMYOTROPHIC LATERAL SCLEROSIS

ALS is a severe type of motor neuron disease (MND) that results in the gradual deterioration of motor neurons in both the brain and the spinal cord [112]. This degeneration eventually leads to the loss of muscle mass throughout the entire body. Like MS, limited studies are exploring the relationship between vitamins and ALS. Nonetheless, like MS, there is a robust correlation between ALS and Vitamin D supplementation [111-113].

PubMed database was used to search the literature, and findings from clinical/preclinical studies on vitamins for managing Amyotrophic Lateral Sclerosis were tabulated in Table **8** [113-120]. The table includes the type of study, findings, outcome, and significance. Vitamin E has been found to prevent the death of NSC-34 motor neurons in ALS. This is achieved through the downregulation of the c-Jun N-terminal kinase (JNK) and p38 MAPK pathways, which cause cell death, as well as the upregulation of the extracellular signal-regulated kinase (ERK) pathway, which promotes cell survival [120]. On the other hand, vitamin D supplementation has been shown to reduce symptoms of muscle weakness and improve motor functional capacity in ALS [116, 117]. However, it does not prevent the final disease outcome [115-117]. In context to clinical findings, vitamin D reduction in ALS patients was found to be beneficial in preventing ALS, protecting motor neurons, and improving muscle weakness [113, 114, 118]. Similarly, vitamin E significantly improved motor functioning in ALS patients [119].

## CONCLUSION

Neurodegenerative diseases can be caused by various factors, and unfortunately, there is currently no known cure for them. However, there are ways to decrease the risk of developing these diseases. Research suggests that specific vitamins may play a role in mitigating neurodegeneration by regulating important molecules and signaling pathways in the brain. This cumulative evidence provides hope for future treatments and future prevention strategies. Based on a comprehensive review of preclinical and clinical findings, this review successfully explored the role of vitamins in preventing neurodegenerative diseases, including AD, PD, MS, HD, and ALS. This review delves into the complex world of vitamins and their potential impact on neurodegenerative diseases, highlighting promising and contentious findings. To gain a more comprehensive understanding of their potential therapeutic benefits, further research is needed, including studies exploring different drug-dose paradigms, diverse humanized animal models, and clinical trials conducted in various locations.

## AUTHORS’ CONTRIBUTIONS

LC wrote the main manuscript text and prepared the table. AB, AKM conceptualized and edited the paper, reviewed the content of the schematic representation, and prepared figures and tables. RR, HS, AKB and SRD reviewed the manuscript and partially assisted with the tables.

## Figures and Tables

**Fig. (1):**
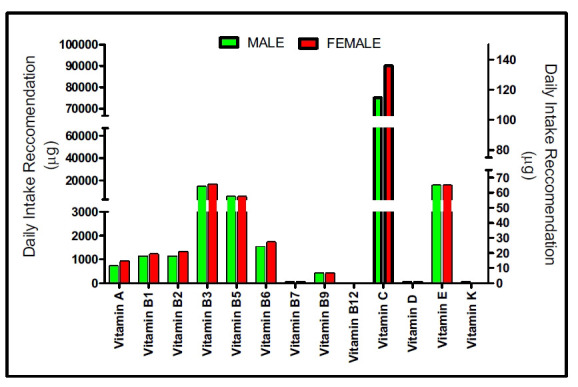
At a glance information on daily vitamin intake recommendation. [WHO Library Cataloguing-in-Publication Data (Vitamin and mineral requirements in human nutrition: report of a joint FAO/WHO expert consultation, Bangkok, Thailand, 21-30 September 1998) & US National Academy of Medicine (https://nap.nationalacademies.org/)].

**Fig. (2):**
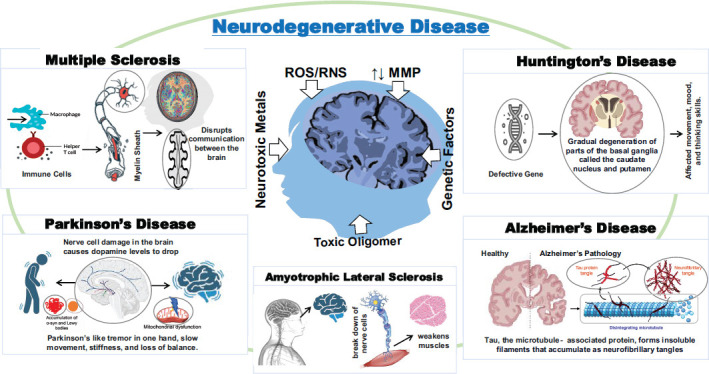
The primary type of neurodegenerative disease (NDD). The schematic representation shows the characteristic features of each disease, their significant sign, and symptoms with etiological interreferences. The reactive oxygen and nitrogen species (ROS, RNS), the effect of neurotoxic oligomers and heavy metals, and heavy genetic predisposition are the critical factors for developing NDD.

**Fig. (3):**
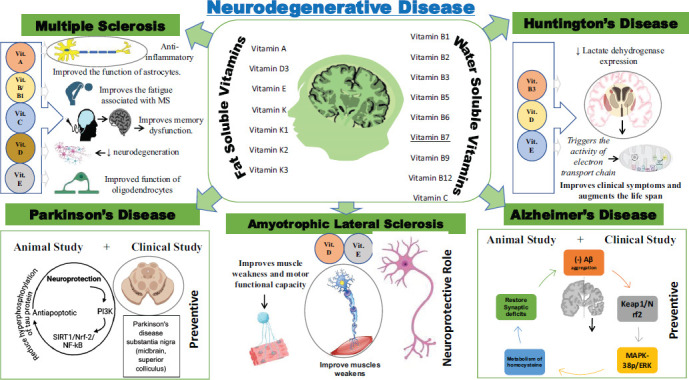
The primary type of neurodegenerative disease. The schematic representation shows the preventive role of different fat and water-soluble vitamins on five primary types of neurodegenerative diseases. The clinical and preclinical findings were detailed and tabulated in Table **2**-**8** .

**Table 1:** Vitamins and their classification. Both water-soluble and fat-soluble vitamins were tabulated here, specifying their common name.

**Classification**	**Vitamin**	**Common Name**
**Water-Soluble** (Not stored in the body. Hence, a continuous exogenous supply is required)	Vitamin B1	Thiamine
	Vitamin B2	Riboflavin
	Vitamin B3	Niacin, Nicotinic Acid
	Vitamin B5	Pantothenic Acid
	Vitamin B6	Pyridoxine and derivatives
	Vitamin B7	Biotin, Vitamin H
	Vitamin B9	Folate, Folacin
	Vitamin B12	Cobalamin
	Vitamin C	Ascorbate, Dehydro-ascorbate
**Fat-Soluble** (Stored in the body; dissolves in fats and oils; are absorbed in the intestine)	Vitamin A	Retinol or all-trans-Retinol
	Vitamin D3	Cholecalciferol
	Vitamin E	Alpha-Tocopherol and 5,7,8-Trimethyltocol
	Vitamin K	Kinadion, Konakion, Mephyton, Monodion
	Vitamin K1	Phytomenadione
	Vitamin K2	Menaquinone
	Vitamin K3	Menadione

**Table 2:** Findings from preclinical studies on vitamins in Alzheimer’s Disease. The literature was searched in PubMed, and the publications were tabulated here, including the type of study, respective findings or outcomes, and significance of the study.

**Vitamin**	**Type of Study**	**Findings/Outcome/ Significance**	**References**
Beta-carotene (a form of Vit A)	Animal Model-Streptozotocin (STZ)-induced AD mouse model.	Levels of toxic B-amyloid fragments were reduced, and cognitive function and oxidative stress improved significantly.	[13]
VitA	*In vitro* - thioflavin T assay	Vitamin A inhibited Aβ aggregation with two Aβ fragments, Aβ1-16 and Aβ25-35.	[14]
Vit C	Animal	Restores behavioural deficits and amyloid-β oligomerization without affecting plaque formation	[15]
9-cis retinoic acid	Animal Model - AD mouse model.	Intranasal administration of 9-cis retinoic acid (9-cis RA) significantly reduced neuronal dysfunction, astrocyte activation, neuroinflammation, and Aβ aggregation. Synaptic deficits were also restored.	[16]
Ascorbic Acid	Animal Model - AlCl_3 _induced rat model of AD.	✓ In a rat model of Alzheimer's disease, ascorbic acid supplementation at a dose of 100 mg/kg for 15 days improved biochemical and behavioural deficits and reduced neuropathological changes. ✓ Ascorbic acid also inhibited the progression of Alzheimer's disease by demonstrating AChE inhibitory and anti-proteolytic activity.	[17]
Vit C	Animal Model - Colchicine-induced neuroinflammation-model for neurodegeneration and cognitive. Impairments.	Lower doses of Vitamin C (200-400 mg/kg BW) protect against colchicine-induced neurodegeneration and cognitive impairments by scavenging free radicals. However, higher doses of Vitamin C (600 mg/kg BW) can lead to oxidative stress, neuroinflammation, and cognitive impairment. Vitamin C, therefore, exhibits dual activity with protective effects at lower doses and damaging effects at higher doses.	[18]
Vit D3	Animal Model - STZ-induced AD model.	Improved cognitive function by inhibiting neuroinflammatory and oxidative stress responses and improved cholinergic function in the mouse model of STZ-induced AD	[19]
Vit D	Animal Model- Age-related cognitive decline in a rat model.	Improved the age-related cognitive decline in a rat model by modulating the activity of proinflammatory cytokines; Vit D also decreased the amyloid burden responsible for cognitive impairment.	[20]
Maxacalcitol (an analog of the active form of Vit D)	Animal Model - LPS-induced rat model of AD.	It improved cognitive function and inhibited neuroinflammation induced by LPS in a rat model of AD through Keap1/Nrf2 and MAPK-38p/ERK signalling pathways.	[21]
Vit D	Animal Model- Mouse model of AD	Vitamin D deficiency can promote AD-like pathology by reducing antioxidative potential and increasing amyloid-beta production, tau phosphorylation, and inflammatory loads. Reducing the deficiency of active vitamin D may lower the risk of Alzheimer's disease and dementia.	[22]
Vit D	Animal Model - D-Galactose-induced memory impairment mice model.	Neuroprotective activity is shown by Vit D *via* SIRT1/Nrf-2/NF-kB signalling pathways.	[23]
α-tocopherol (a type of Vit E)	Animal model - Wistar rats infused with α-tocopherol	Vitamin E prevents learning and memory deficits.	[24]
Multivitamin	Animal Study - A Retrospective study.	A retrospective study linked Higher folate concentration to improved cognitive performance. Folate deficiency is associated with worse cognitive performance and hyperhomocysteinemia.	[25]
Multivitamin	Animal Study - Hypoxia-induced memory deficits mouse model	Vitamins B6, B12, folate, and choline may protect the brain in low-oxygen conditions and improve memory. These vitamins reduce hyperphosphorylation of tau and homocysteine levels. A multivitamin approach can alleviate memory deficits caused by hypoxia.	[26]
Vitamin K2	Animal Model - Aβ induced neurotoxicity model in animal	Prevented the Aβ induced neurotoxicity by the phosphatidylinositol 3-kinase (PI3K) associated signalling pathway.	[27]

**Table 3:** Findings from clinical studies on vitamins for Alzheimer’s Disease. The literature was searched in PubMed, and the publications were tabulated here, including the type of study, respective findings or outcomes, and significance of the study.

**Vitamin**	**Type of Study**	**Findings/Outcome/ Significance**	**References**
Vit A, B, C	Clinical Study- AD patients were compared to healthy volunteers.	Reported lower levels of Vit A, Vit B, and Vit C in the blood serum of AD patients when compared to healthy individuals (with intact neurocognitive function)	[28]
Vit A, B, C, D, E, K	Clinical Study -Aeta-analytical study.	Reduced serum levels of Vit A and Vit B were observed in AD patients. The regulatory role of Vit C, Vit D, Vit E, and Vit K in AD patients was demonstrated.	[16, 29] [30, 31]
Vit A, C, E	Clinical Study- AD (Dementia) patients were compared to healthy volunteers.	Reduced levels of Vit A, Vit C, and Vit E were observed in dementia patients compared to healthy controls.	[32, 33]
Vit C, E, Vit B	Clinical Study - A cross-sectional and prospective study.	A combination of vitamin C and vitamin E supplementation reduced the prevalence and incidence of Alzheimer's disease. No association was found between vitamin B intake and Alzheimer's disease.	[34]
Retinoids	Clinical Study -Preventive Approach.	Cognitive function in US women was significantly improved by long-term treatment with retinoids.	[35]
Vit B2, B6, B9, B12	Clinical Study - Preventive and Etiological Study.	✓ Several types of B vitamins (Vit B9, Vit B12, Vit B6, and Vit B2) are involved in the metabolism of homocysteine; ✓ Elevated levels of total plasma homocysteine lead to cognitive impairments, which may ultimately lead to dementia. ✓ Studies have demonstrated that Vit B supplementation lowers total homocysteine in the treatment of cognitive decline.	[36] [37, 38], [39] [40, 41], [42]
Vit B	Clinical Study - Unbiased analysis.	Supplementing with Vitamin B reduces AD pathology, characterized by cerebral grey matter atrophy, by decreasing the total homocysteine level in serum plasma.	[37]
Vit B (supplementation)	Clinical Study-Meta-analysis.	A meta-analysis of randomized controlled trials found no improvements in cognitive impairment with therapies that reduce total homocysteine through vitamin B supplementation.	[43-45]
Multivitamin supplement containing Vitamins B6, B12, and B9 in addition to Acetylcholinesterase Inhibitor treatment	Clinical Study - Randomized, double-blind, and placebo-controlled study of Taiwanese AD patients.	The results showed a decrease in serum homocysteine concentration but did not indicate any positive effects on the cognitive function or daily living activities of the patients with Alzheimer's disease.	[46]
Vit B9, B6, B12	Clinical Study- On mild to moderate AD patients.	A high dose of B vitamins (Vit B9, Vit B12, Vit B6), while effectively lowering homocysteine levels, did not affect cognition in individuals with mild to moderate AD.	[47]
Vit B	Clinical Study- Randomized placebo-controlled trial in older patients.	Vitamin B prevented cognitive decline, as demonstrated by a randomized placebo-controlled trial.	[48]
Vit B	Clinical Study -Secondary data from an RCT and its analysis.	A 2-year vitamin B treatment has no significant effect on elevated homocysteine levels and cognitive performance based on secondary data from an RCT.	[49]
Vit B12, Folate	Clinical Study- Cross-sectional study.	Higher vitamin B12 and folate exhibited potent therapeutic activity and improved cognitive performance in a cross-sectional study on AD.	[50]
Vit B12 and serum folic	Clinical Study - Turkish-based multicentred study.	The screening of pre-symptomatic AD cannot be diagnosed by measuring the serum folic and vitamin B12 levels.	[50]
Vit B1	Clinical Study - AD affected individual study.	Significant cognitive impairment with progressive dementia has been improved by the supplementation of thiamine in affected individuals.	[51]
Vit B12	Clinical Study - Single Centre study on AD patients.	Vitamin B12 inhibits the tau fibrillization and formation of the neurofibrillary tangle; thus, VitB12 prevents the tau aggregation and neurofibrillary tangle formation that might progress the severity of AD.	[52]
Vit A, B2, C	Clinical Study- Havana, Cuba-based study on AD patients.	Low levels of vitamins A, C, and B2 were found in older adults with Alzheimer's disease, while their homocysteine levels were higher. However, vitamin B12, folic acid, and thiamine levels did not show any relation to cognitive impairment. The study suggests that vitamin deficiencies might impact homocysteine metabolism in Alzheimer's and MCI patients.	[53]
Folic Acid	Clinical Study- A Trial conducted in China	Folic acid shows anti-inflammatory solid activity and prevents neurodegeneration in AD.	[54]
Vit B12	Clinical Study - Korean population-based study.	Cognitive function is affected by the B12 levels, as suggested by an elderly Korean population-based study.	[55]
Vit E	Clinical Study - AD patients	Vit E is essential in improving cognitive function and memory deficits.	[25]
Vit E	Clinical Study - On Cognitively impaired (MCI) patients (early stage of AD).	The impact of VitE in conjunction with donepezil supplementation for mild cognitively impaired (MCI) patients (early stage of AD) was reported, and no added benefit was found from Vit E supplementation.	[13]

**Table 4:** Findings from preclinical studies on Parkinson’s Disease considering the role of vitamins in its prevention. The literature was searched in PubMed, and the publications were tabulated here, including the type of study, respective findings or outcomes, and significance of the study.

**Vitamin (s)**	**Type of Study**	**Findings/ Outcome/ Significance**	**References**
Retinol	Animal Study - 6-hydroxydopamine intoxicated Wistar rat model.	✓ There is no effect of oral retinol supplementation in the 6-hydroxydopamine intoxicated Wistar rat model. ✓ Receptor-based therapeutic approaches have been found beneficial in preventing the progression of PD.	[68]
Lycopene (Carotenoid)- Vitamin A Precursor	Animal Study - MPTP-induced Parkinsonian mouse model.	✓ Exhibits its effectiveness in the MPTP-induced Parkinsonian mouse model. ✓ The lycopene treatment reversed physiological anomalies, oxidative stress, neurochemical abnormalities, and apoptosis; ✓ Lycopene shows anti-apoptotic and antioxidative properties in this PD mouse model.	[69]
Lycopene- Vitamin A Precursor	Animal Study - Rotenone-induced PD model	Protects the cognitive decline in the rotenone-induced PD model.	[70]
Vit C	Animal Study- UCH-L1 gene knockdown PD model in Drosophila.	Mitigated the PD-like phenotype of dopaminergic neuron degeneration and locomotor deficits in a UCH-L1 gene knockdown Drosophila PD model.	[71]
Vit C	Animal Model - UCH-L1 gene knockdown Drosophila PD model)	Explored the dose-dependent preventive effects of vitamin C using this knockdown fly model.	[72]
Vit D	Animal Study - Hemi- parkinsonian rat model	Vit D protects the death of dopaminergic neurons by inhibiting oxidative stress and neuroinflammation.	[73]
Vit E	Animal Model - Mouse knockdown model	Vit E supplementation significantly improved PD-like symptoms and associated pathology regarding mitochondrial abnormalities and synaptic impairment in a PD knockdown model.	[74]
Vit E	Animal Study - Zitter mutant rat model of PD.	Chronic intake of VitE (500 mg/kg diet) lowers the death of dopaminergic neurons in the substantia nigra of the zitter mutant rat model of PD.	[75]
Vit E	Animal Study - PD progression induced by intra-striatal injection of 6-OHDA.	In the PD rat model, PD progression induced by intra-striatal injection 6-OHDA was ameliorated by Vit E treatment.	[76]
Vit E	Animal Study - Early rat PD model induced by unilateral intra-striatal 6-hydroxydopamine.	Repeated intramuscular administration of vitamin E (24 I.U./kg, i.m) offers significant neuroprotective properties in the model (on the nigrostriatal dopaminergic neurons in the early unilateral model of PD).	[77]
Alpha-tocopherol	Animal Study -Unilateral 6-OHDA model in the animal.	Exhibited a neuroprotective activity in the model.	[78]

**Table 5:** Findings from clinical studies in Parkinson’s Disease (PD) considering the role of vitamins in preventing PD. The literature was searched in PubMed, and the publications were tabulated here, including the type of study, respective findings or outcomes, and significance of the study.

**Vitamin**	**Type of Study**	**Findings/ Outcome/ Significance**	**References**
Vit A	Clinical Study - This biomarker-based clinical study assessed the therapeutic impact of Vit A in the frontal lobe cortex.	This demonstrated high concentrations of Vit A and its derivatives in the human post-mortem frontal lobe cortex. The frontal lobe cortex showed an age-related decline in retinol and its derivatives compared to the occipital cortex.	[79]
Vit A, C, E	Clinical Study- A Singaporean Chinese cohort-based study.	Suggested no correlation between dietary antioxidants, such as carotenoids and vitamins (vitamins A, C, and E), and the risk of developing PD.	[80]
Vit B9	Clinical Study - PD *versus* average healthy comparison.	No significant change was observed regarding Vit B9 concentration in PD patients *versus* normal healthy controls.	[81, 82]
Vit B1	Clinical Study - An open-level study.	Parkinsonian symptoms become reversed significantly by the intramuscular administration of a high dose of thiamine to PD patients without any side effects.	[83, 84]
Vit B1, B9	Clinical Study - Study on PD patients with olfactory dysfunction.	PD patients with olfactory dysfunction observed 2-8 years before displaying symptoms demonstrated low dietary Vit B1 and Vit B9 density.	[83, 84]
Vit C	Clinical Study - Study on elderly PD patients.	Ascorbic acid (200 mg) significantly improves levodopa's absorption (100 mg levodopa and 10 mg carbidopa) in elderly PD patients.	[85]
Calcitriol (1,25-dihydroxy vitamin D3)	Clinical Study- Comparison of age-matched healthy controls with PD patients.	Compared to age-matched healthy controls, PD patients have a much lower concentration of calcitriol in their blood plasma, which may be related to bone health and fracture risk.	[86, 87]
Vit D	Clinical Study - A meta-analysis)	There is an inverse relationship between Vit D level and risk and severity of PD in 2866 PD patients; there is a limited response to Vit D supplementation (≥400 IU/day) in early PD patients.	[88, 89]
Vit E	Clinical Study - Single-centre Study on PD patients.	The effect of Vit E was age- and sex-independent and showed an inverse relation between Vit E intake and PD occurrence; diets rich in Vit E may minimize the risk associated with PD.	[74]
Vit E	Clinical Study - A study on early untreated PD patients.	High-dose vitamin E (2000 IU vitamin E orally per day) treatment enhanced its concentration in the cerebrospinal fluid (CSF) of an early untreated PD patient; high CSF and a high brain concentration of alpha-tocopherol show a protective effect on PD patients.	[90]
Vit E	Clinical Study- Single-center study on PD patients.	Recommended daily multivitamin supplementation should contain at least 30 IU of alpha-tocopherol instead of 400 IU of alpha-tocopherol in the affected individuals; this combination showed an improved therapeutic response in PD patients.	[91]
Vit C, E, Carotenoids	Clinical Study - Single-center study.	Utilizing a multivitamin strategy consisting of vitamin E, C, and carotenoids does not appear to reduce the risk of Parkinson's disease. Instead, increasing the intake of dietary supplements rich in vitamin E may offer improved therapeutic potential for reducing the risk of PD. However, it is essential to note that excessively high doses of vitamin E may result in a loss of its therapeutic effects.	[92]

**Table 6:** Role of different vitamins in Huntington’s Disease. The literature was searched in PubMed, and the publications were tabulated here, including the type of study, finding, outcome, and significance.

**Vitamin**	**Type of Study**	**Findings/Outcome/ Significance**	**References**
Nicotinamide (Vit B3)	Animal Study- Chemical-induced rat model of HD	Nicotinamide improved motor functioning, prevented the progression of neurodegeneration, and exhibited potent neuroprotective activity in a rat model. It also decreased tissue degradation by reducing the expression of lactate dehydrogenase.	[93]
Calcitriol (Vit D)	Animal Study - Transgenic mouse HD model	Supplementation of calcitriol significantly improved clinical symptoms and augmented the life span. Thus, serum calcitriol is positively related to the treatment of HD.	[94]
CoQ10 and Vit E	Animal Study - NPA-induced rat model of HD	This study explored CoQ10 and VitE's synergistic effects on HD models of NPA-induced rats, which show reduced energy balance. CK levels, a brain disease energy biomarker, were elevated in the HD model, while CoQ10, ATP, and electron transport chain activity were reduced. CoQ10 and VitE supplementation successfully reversed these abnormalities.	[95]
α-tocopherol (a significant constituent of Vit E)	Clinical Study - A localized and limited clinical trial	Slowed the progression of motor abnormalities associated with HD.	[96]

**Table 7:** Findings from clinical/animal studies on vitamins preventing Multiple Sclerosis. The literature was searched in PubMed, and the publications were tabulated here, including the type of study, finding, outcome, and significance.

**Vitamin**	**Type of Study**	**Findings/Outcome/ Significance**	**References**
Vit A	Clinical Study - A single-centre pilot study	Vit A exhibited anti-inflammatory and antioxidant activity in the brain, and Vit A serum levels were reduced in MS patients.	[99-101]
Vit A	Clinical Study - A single-centre pilot study	Improved the function of astrocytes, led to remyelination, and suppressed immune function in MS patients.	[78]
Vit A	Clinical Study - A single-centre pilot study	In contrast, results from one study demonstrated no correlation between serum Vit A concentration and the progression of MS.	[102]
Vit B	Clinical Study - A single-centre pilot study	Some studies have found that Vit B levels are reduced in MS patients, while others show no relation between Vit B levels and MS.	[103]
Vit B1 (Thiamine)	Clinical Case Report Study	High-dose thiamine (VitB1) therapy improved the fatigue commonly associated with MS.	[104]
Vit C	Clinical Study - A single-centre pilot study	As compared to a healthy individual, MS patients exhibit reduced levels of Vit C.	[105-107]
Vit C	Animal Study - A rat model of MS.	Direct intrahippocampal injections of Vit C in a rat model of MS improved memory dysfunction on passive avoidance learning.	[97]
Vit D	Clinical Study - Observational Study on Patients	Observational studies and clinical trials have shown that a reduced level of Vit D in the blood is a risk factor for developing MS.	[100, 108, 109]
Vit D	Clinical Study - A single-centre pilot study	Supplementation with Vit D enhanced blood perfusion, which supports tissue oxygenation and, therefore, reduced neuroinflammation and neurodegeneration.	[110]
Vit E	Clinical Study - A single-centre pilot study & a model of neutrophilic airway inflammation in rats.	Showed the improved function of oligodendrocytes and inhibition of factors related to the necrosis process in MS.	[111]
Vit C	Animal Study - The lesion was induced by intrahippocampal injection of ethidium bromide in rats.	✓ It causes demyelination in the neurons *via *disruption in collagen synthesis. ✓ Deficiency increases the oxidative stress in the neurons and decreases the expression of the antioxidant molecules in the brain's neurons.	[97]
Vit C	Clinical Study -	✓ Compared to a healthy individual, MS patients exhibit reduced levels of Vit C. ✓ It also showed a Neuroprotective effect.	[105-107, 112]
Vit D	Animal Study - ✓ Juvenile/adolescent rat model of MS compared to adult-aged animals.	✓ Vit D supplementation was more efficacious at ameliorating MS-like neuroinflammation. ✓ Protective activity that usually depends on the patient’s developmental stage.	[98]

**Table 8:** Findings from clinical/preclinical studies on vitamins for managing Amyotrophic Lateral Sclerosis. The literature was searched in PubMed, and the publications were tabulated here, including the type of study, finding, outcome, and significance.

**Vitamin**	**Type of Study**	**Findings/Outcome/ Significance**	**References**
Vit D	Clinical Study - Multinational case-control study	The active form of vitamin D is reduced in ALS patients and animal models of ALS.	[113]
Vit D	Clinical Study - Single centre study & *in vitro* Assay	Vit D was shown to be protective in motor neurons *in vitro*, and plasma levels of Vit D were directly correlated to the severity of the disease in ALS patients.	[114]
Vit D	Animal Study - Transgenic mouse models of ALS.	Vit D supplementation reduced symptoms of muscle weakness and improved motor functional capacity but did not prevent the final disease outcome.	[115-117]
Vit D	Clinical Study - Single-centre study	No reduction in Vit D levels and no benefit from Vit D supplementation for improving the prognosis of this disease was observed.	[118]
Vit E	Clinical Study - Single-centre study	✓ Individuals who did not take the regular dose of Vit E exhibited early death as compared to those who regularly took Vit E supplementation. ✓ VitE also significantly improved motor functioning in ALS patients.	[119]
Vit E	Cell culture Study	Vit E prevented the death of NSC-34 motor neurons in ALS through the downregulation of the c-Jun N-terminal kinase (JNK) and p38 MAPK pathway (cell death) and upregulation of the extracellular signal-regulated kinase (ERK) pathways (cell survival).	[120]

## LIST OF ABBREVIATIONS

AD: Alzheimer's Disease
ALS: Amyotrophic Lateral Sclerosis
DRI: Dietary Reference Intakes
HD: Huntington's Disease
MCI: Mild Cognitive Impairment
MND: Motor Neuron Disease
MS: Multiple Sclerosis
NDDs: Neurodegenerative Diseases
PD: Parkinson's Disease
RNI: Reference Nutrient Intakes
RNS: Reactive Nitrogen Species
ROS: Reactive Oxygen Species
